# What are the predictors, barriers and facilitators to effective management of acute pain in children by ambulance services? A mixed-methods systematic review protocol

**DOI:** 10.29045/14784726.2018.09.3.2.22

**Published:** 2018-09-01

**Authors:** Gregory Adam Whitley, A. Niroshan Siriwardena, Pippa Hemingway, Graham Richard Law

**Affiliations:** University of Lincoln; East Midlands Ambulance Service NHS Trust; University of Lincoln; University of Nottingham; University of Lincoln

**Keywords:** child, emergency medical services, pain

## Abstract

**Introduction:**

The management of pain is complex, especially in children, as age, developmental level, cognitive and communication skills and associated beliefs must be considered. Without effective pain treatment, children may suffer long-term changes in stress hormone responses and pain perception and are at risk of developing posttraumatic stress disorder. Pre-hospital analgesic treatment of injured children is suboptimal, with very few children in pain receiving analgesia. The aim of this review is to identify predictors, barriers and facilitators to effective management of acute pain in children by ambulance services.

**Methods:**

A mixed-methods approach has been adopted due to the research question lending itself to qualitative and quantitative inquiry. The segregated methodology will be used where quantitative and qualitative papers are synthesised separately, followed by mixed-methods synthesis (meta-integration). We will search from inception: MEDLINE, CINAHL and PsycINFO via EBSCOHost, EMBASE via Ovid SP, Web of Science and Scopus. The Cochrane Library, the Joanna Briggs Institute, PROSPERO, ISRCTN and ClinicalTrials.gov will be searched. We will include empirical qualitative and quantitative studies. We will exclude animal studies, reviews, audits, service evaluations, simulated studies, letters, Best Evidence Topics, case studies, self-efficacy studies, comments and abstracts. Two authors will perform full screening and selection, data extraction and quality assessment. GRADE and CERQual will determine the confidence in cumulative evidence.

**Discussion:**

If confidence in the cumulative evidence is deemed *Moderate, Low or Very Low*, then this review will inform the development of a novel mixed-methods sequential explanatory study which aims to comprehensively identify predictors, barriers and facilitators to effective pain management of acute pain in children within ambulance services. Future research will be discussed among authors if confidence is deemed *High*.

Systematic Review Registration: PROSPERO: CRD42017058960.

## Background

Pain is ’an unpleasant sensory and emotional experience associated with actual or potential tissue damage, or described in terms of such damage’ (International Association for the Study of Pain, 1994). Acute pain is defined as pain that lasts less than 12 weeks (British Pain Society, 2018). According to the World Health Organization (2015) and Lohman, Schleifer, and Amon (2010), all countries must provide pain treatment medication as a core obligation under the right to health. The management of pain is complex, especially in children, as age, developmental level, cognitive and communication skills and associated beliefs must be considered (Srouji, Ratnapalan, & Schneeweiss, 2010). Pain can have psychological, physical and social consequences which impact on quality of life (Lohman et al., 2010). Without effective pain treatment, children may suffer long-term changes in stress hormone responses and pain perception (Finley, Franck, Grunau, & von Baeyer, 2005) and are at risk of developing posttraumatic stress disorder (Saxe et al., 2001; Sheridan et al., 2014).

Pre-hospital analgesic treatment of injured children is suboptimal (Samuel, Steiner, & Shavit, 2015), with very few children in pain receiving analgesia (Hennes, Kim,&Pirrallo, 2005; Lerner et al., 2014; Shaw, Fothergill, & Virdi, 2015; Swor, McEachin, Seguin, & Grall, 2005; Whitley & Bath-Hextall, 2017). One US study (Lerner et al., 2014) found that from 55,642 pre-hospital patients aged <19 years, 42.1% suffered a traumatic injury or pain, yet only 0.3% received analgesia. A recent UK study found that of injured children (<18 years) who reported pain (n = 7483), 38.8% received no treatment (Whitley & Bath-Hextall, 2017), therefore there is a real need to identify barriers to effective pain management.

Studies from Ireland (Murphy et al., 2014), the United States (Williams, Rindal, Cushman, & Shah, 2012) and Canada (Rahman et al., 2015) have identified barriers and facilitators to pre-hospital pain management in children. One of these studies lacked transferability because it did not reflect the wider paramedic community, interviewing only advanced paramedics (Murphy et al., 2014), and transferability was also an issue with the other studies due to differing emergency medical service and educational systems (Rahman et al., 2015; Williams et al., 2012).

Other studies have identified a number of predictors associated with pre-hospital pain management processes for children (Browne et al., 2016; Hewes, Dai, Mann, Baca, & Taillac, 2017; Lord, Jennings, & Smith, 2016; Whitley et al., 2017). There are no systematic reviews to date that identify known predictors, barriers and facilitators of pre-hospital pain management in children.

The following review question was identified: What are the predictors, barriers and facilitators to effective management of acute pain in children by ambulance services? Considering the range of qualitative and quantitative studies which seek to address the question, it seemed appropriate to perform a mixed-methods systematic review in order to reach a consensus of all predictors, barriers and facilitators of pre-hospital management of acute pain in children.

## Methods

### Aim

We aim to review the evidence which identifies the predictors, barriers and facilitators to effective management of acute pain in children by ambulance services.

### Design

This mixed-methods systematic review protocol is based on the guidance of the Joanna Briggs Institute (2014), the ’Preferred Reporting Items for Systematic Review and Meta-Analysis Protocols’ (PRISMA-P) guidelines (see Supplementary 1) (Shamseer et al., 2015) and Boland, Cherry, and Dickson (2017). Due to the relative infancy of mixed-methods systematic review methodology, a number of designs exist: realist synthesis along with segregated, integrated and contingent methodologies (Joanna Briggs Institute, 2014). Based on the early work of Thomas et al. (2003, 2004), whose methodology was later cited in Sandelowski, Voils, and Barroso (2006), the segregated methodology seemed appropriate to this study as qualitative data exploring barriers and facilitators will be synthesised with quantitative data identifying predictors. The segregated design synthesises qualitative and quantitative data separately, followed by a mixed-methods synthesis (meta-integration) (Sandelowski et al., 2006). This differs from the integrated design where the synthesis is combined (assimilation) and the contingent design where multiple research questions are addressed by synthesising one study type at a time, in a stepwise fashion, with each synthesis leading to a further research question (Sandelowski et al., 2006). See Figure 1 for the modified segregated methodology diagram of procedures.

**Figure:**
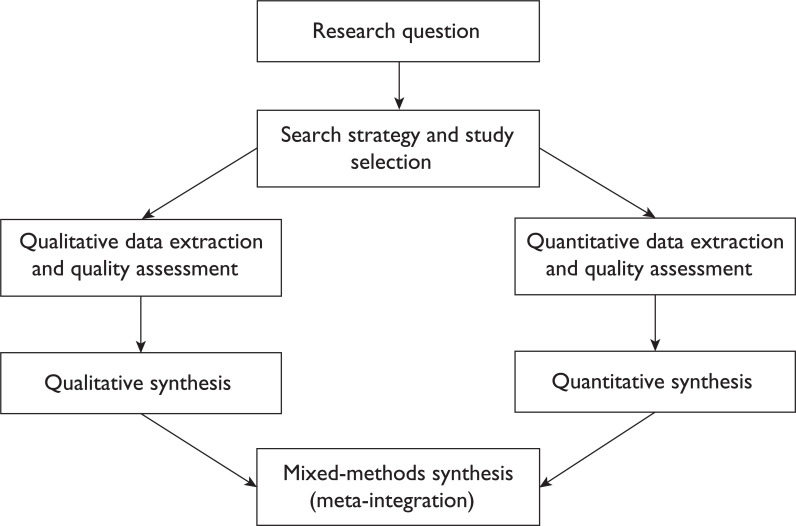
Figure 1. Diagram of procedures for the mixed-methods systematic review – modified segregated approach.

Adapted from Sandelowski et al. (2006), cited in Joanna Briggs Institute (2014).

### Inclusion criteria

No language restrictions will be placed on the review.

#### Qualitative studies

- Participants. Ambulance service/emergency medical service staff; patients (aged <18 years); relatives.
- Phenomena of interest. This review will consider studies that identify barriers and facilitators of pain management in children (aged <18 years) treated by ambulance services.
- Context. All international pre-hospital emergency medical services/ambulance services.
- Types of study. Qualitative designs including but not limited to phenomenology, grounded theory, ethnography and generic qualitative approach.

#### Quantitative studies

- Participants. Ambulance service/emergency medical service staff; patients aged <18 years, suffering acute pain and attended by ambulance service/emergency medical service staff.
- Phenomena of interest. This review will consider studies that identify predictors associated with effective or ineffective management of acute pain in children within pre-hospital emergency medical services/ambulance services.
- Context. All international pre-hospital emergency medical services/ambulance services.
- Types of study. Quantitative approaches included but not limited to interventional studies, observational studies (cohort and case control), cross-sectional studies and surveys.

#### Multi-methods studies

- Must meet qualitative and/or quantitative inclusion criteria as above. Multi-methods studies included will have their qualitative and/or quantitative data extracted into their respective arms of the review.

### Exclusion criteria

Animal studies, reviews, audits, service evaluations, simulated studies, letters, Best Evidence Topics (BestBETs), case studies, self-efficacy studies, comments and studies only reporting an abstract will be excluded. Quantitative studies including children and adults where the child specific data cannot be extracted will be excluded.

Relevant studies that do not conform to the qualitative, quantitative or multi-methods inclusion criteria will not be included in the main synthesis but will be discussed narratively. Given the segregated approach taken, mixed-methods studies are not suitable for inclusion in the main synthesis due to their inherent integration, but will be discussed narratively.

### Search strategy

The following databases will be searched from inception:

- MEDLINE via EBSCOHost
- EMBASE via Ovid SP
- CINAHL via EBSCOHost
- PsycINFO via EBSCOHost
- Web of Science
- Scopus

The Cochrane Library, Joanna Briggs Institute and PROSPERO will be searched for relevant systematic reviews. Relevant systematic reviews will have their reference list searched for additional studies. Reference lists of included studies will be searched and leading authors in the field will be contacted regarding unpublished/grey literature. Google Scholar and Open Grey will be used to identify articles not indexed in the major databases.

Trial registries including ISRCTN and ClinicalTrials.gov will be searched for any relevant interventional studies.

### Search terms

The following keywords will be used:

(Infant* OR Child* OR Pediatric* OR Paediatric* OR Adolescen*) AND (Ambulance* OR "Emergency Medical Service*" OR Prehospital OR Pre-Hospital OR "Out of Hospital" OR Paramedic*) AND (Pain OR Analgesi* OR Oligoanalgesia)

If appropriate, keywords will be adapted according to database subject headings. See Figure 2 for draft search strategy for EMBASE via Ovid SP.

**Figure:**
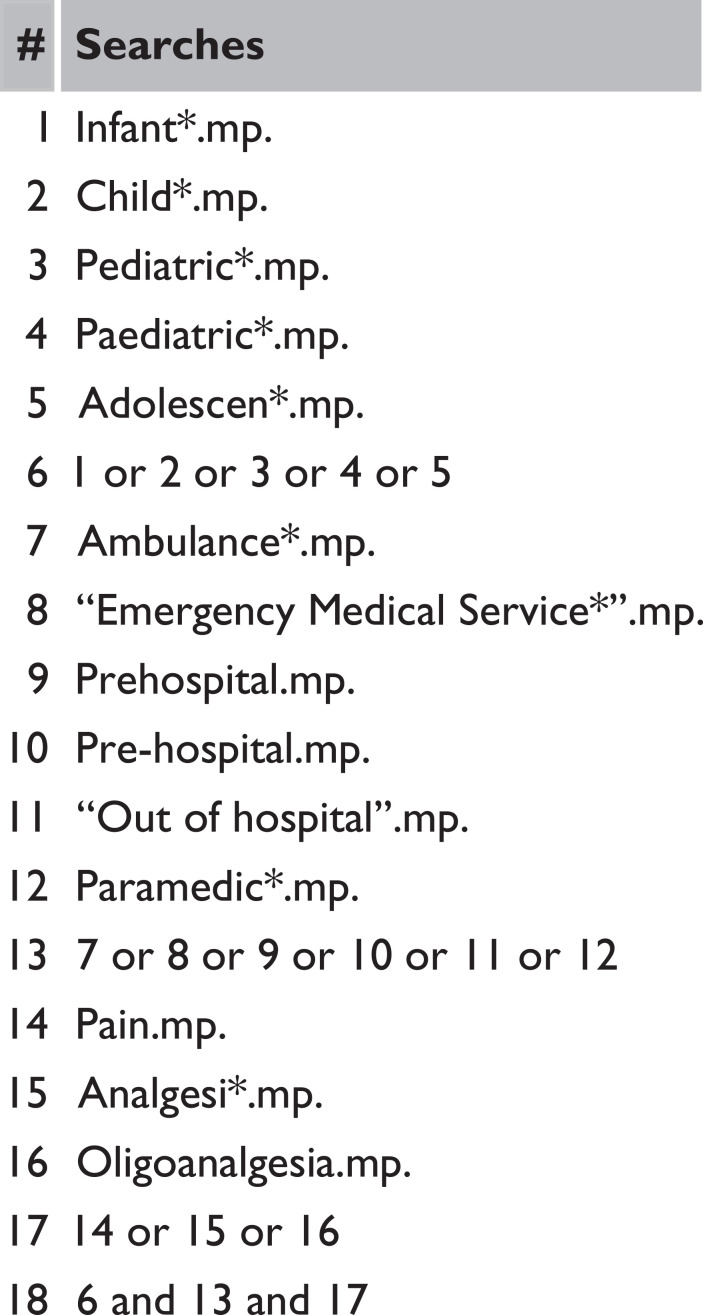
Figure 2. Worked search for EMBASE via Ovid SP.

### Screening and selection

Studies found using the search strategy will be imported into Endnote X8 where duplicates will be removed. Studies will then be sifted by title and abstract followed by a full-text sift according to the inclusion and exclusion criteria. The primary reviewer (GAW) will undertake the screening and selection process which will be duplicated in full by a secondary reviewer (ANS).

### Data extraction

Data will be extracted from studies meeting the inclusion criteria using a standardised extraction tool, to include details about the study methods, population characteristics, outcomes of significance and recommendations among other fields. Outcomes of significance will include key themes arising, identified barriers and facilitators and predictors found to influence the pain management process. A pilot extraction will take place in order to refine the data extraction criteria. Once finalised, the extraction will be performed by two reviewers (GAW and ANS) and disagreements will be settled through discussion or the involvement of a third reviewer (PH) to enable consensus to be reached.

### Quality assessment

Quality assessment of included studies will be performed in duplicate by GAW and ANS. Both authors will determine the level of risk of each study using the appropriate appraisal tool (see below) and associated guidance. The results of this process will be displayed in a ’risk of bias’ table. Studies deemed at high risk of bias will either be adjusted during the synthesis or removed from inclusion if adjustment is not possible.

The following quality assessment approaches will be used to assess the quality and risk of bias of each study meeting the inclusion and exclusion criteria.

#### Qualitative

Quality assessment will follow the Cochrane Quality and Intervention Methods Group guidance (Hannes, 2011), specifically assessing: 1) filtering, only including empirical qualitative studies with descriptions of the methodology chosen, sampling strategy, data collection procedures, type of data analysis; 2) technical appraisal, via a tool such as the Critical Appraisal Skills Programme Qualitative Research Checklist (CASP, 2013); and, where appropriate, 3) theoretical appraisal, focusing on the research paradigm used, as proposed by Popay, Rogers, and Williams (1998: 348), with the assessment of ’evidence of theoretical and conceptual adequacy’.

#### Quantitative

- Interventional studies. *Cochrane handbook for systematic reviews of interventions* (Higgins & Green, 2011), with specific assessment of random sequence generation, allocation concealment, blinding, intention to treat, incomplete outcome data, selective reporting and other sources of bias.
- Cross-sectional studies. The appraisal tool for cross-sectional studies (AXIS tool) (Downes, Brennan, Williams, & Dean, 2016).
- Cohort studies. The Scottish Intercollegiate Guidelines Network (SIGN) methodology checklist for cohort studies (Scottish Intercollegiate Guidelines Network, 2017).
- Case-control studies. The Scottish Intercollegiate Guidelines Network (SIGN) methodology checklist for case-control studies (Scottish Intercollegiate Guidelines Network, 2017).
- Survey studies. Best Evidence Topics critical appraisal worksheet for surveys (BestBETs, 2018).

### Synthesis/analysis

Following the methods of the Joanna Briggs Institute (2014) and Sandelowski et al. (2006), a separate analysis of quantitative and qualitative data will be performed, followed by a final mixed-methods synthesis. This negates the need for Bayesian methods for the mixed-methods synthesis, where quantitative and qualitative papers are assigned a numerical value allowing aggregation of data (Crandell, Voils, Chang, & Sandelowski, 2011).

#### Qualitative synthesis

Thematic synthesis as described by Thomas and Harden (2008) from the Evidence for Policy and Practice Information and Co-ordinating Centre (EPPI-Centre), UK, will be used to synthesise eligible qualitative studies. This process involves three steps: 1) coding text; 2) developing descriptive themes; and 3) generating analytical themes.

#### Quantitative synthesis

##### Assessment of heterogeneity

Heterogeneity will be assessed by comparing factors such as population age and study type. The *I*^2^ statistic will be used to determine heterogeneity. Given a reasonable level of heterogeneity (*I*^2^ ≤50%), a meta-analysis will be performed. Where substantial heterogeneity is found, a narrative analysis will be performed.

##### Measurement of treatment effect

If sufficient studies are available with a reasonable level of heterogeneity, a meta-analysis will be performed. The outcome measure will be effective pain reduction (pain score reduction of ≥2 out of 10 on the numeric pain rating scale or Wong-Baker faces scale), with potential risk factors including, but not limited to: age, gender, injury type, distance to hospital and socio-economic status. Where a meta-analysis is not feasible, a narrative analysis will be performed.

##### Subgroup and sensitivity analysis

A sensitivity analysis will be performed given enough studies and where a number of studies are identified as a ’high risk of bias’.

##### Mixed-methods synthesis (meta-integration)

Once the initial quantitative analysis, or meta-analysis if appropriate, and qualitative meta-synthesis have been performed, a final mixed-methods synthesis using triangulation (Sandelowski et al., 2006) will identify data that either confirm or refute each other. Following the methods of Frantzen and Fetters (2016), this meta-integration will be displayed in tabular format to illustrate the complex inter-relational connections.

### Missing data

An attempt will be made to contact the corresponding author of articles where missing data exist. Where missing data cannot be acquired, the impact on the quality of the study will be discussed.

### Meta-bias(es)

Included interventional studies will be assessed for reporting bias by searching for a published protocol or registration with a clinical trials registry. Where outcomes are specified in the protocol, but not reported in the final report, a risk of bias will be suspected.

### Confidence in cumulative evidence

The Grading of Recommendations Assessment Development and Evaluation (GRADE) approach (Guyatt et al., 2008) will be used to assess the quantitative synthesis. The following domains will be assessed: risk of bias, consistency, directness, precision and publication bias.

The Confidence in the Evidence from Reviews of Qualitative Research (CERQual) (Lewin et al., 2015) approach will be used to guide the overall assessment of the qualitative synthesis. The four components of CERQual are: methodological limitations, relevance, coherence and adequacy of data.

Overall quality will be adjudicated as *High* (further research unlikely to change conclusions), *Moderate* (further research may change conclusions), *Low* (further research likely to change conclusions) and *Very Low* (very uncertain about current conclusions).

## Discussion

If overall confidence in the cumulative evidence is deemed *Moderate*, *Low* or *Very Low*, then this review will inform the development of a mixed-methods sequential explanatory study which aims to comprehensively identify predictors, barriers and facilitators to effective pain management of acute pain in children within ambulance services. The proposed mixed-methods sequential explanatory study is novel and, in combination with the results of this mixed-methods systematic review, will be used to inform the development of an educational intervention and/or further research.

Should this confidence be deemed *High*, then further research within this area will be reviewed by the authors.

## Acknowledgements

We would like to thank Marishona Ortega, information scientist for the School of Health and Social Care at the University of Lincoln, for her input in developing the search strategy. Viet-Hai Phung also provided guidance during the development of this protocol.

## Author contributions

GAW is the guarantor. GAW was responsible for conception. All authors have substantially contributed to the design and development of this protocol. GAW drafted the initial manuscript. All authors agreed to the final version of the manuscript.

## Conflict of interest

None declared.

## Funding

The research was funded by the NIHR Collaboration for Leadership in Applied Health Research and Care East Midlands (CLAHRC EM). The views expressed are those of the author(s) and not necessarily those of the NHS, the NIHR or the Department of Health and Social Care.

The NIHR CLAHRC EM has not been involved in the development of this protocol and will not be involved in the collection, analysis, interpretation or publication phase of this review.
